# The Use of 360° VR Video, Educational Videos, and High‐Fidelity Physical Models in Teaching Breech Birth ‐ A Pilot Feasibility Study

**DOI:** 10.1111/ajo.70040

**Published:** 2025-05-07

**Authors:** Lin Yang, Andrew Bisits

**Affiliations:** ^1^ Obstetrics and Gynaecology The Sutherland Hospital Caringbah New South Wales Australia; ^2^ Adjunct Associate Lecturer, Office of Medical Education, Faculty of Medicine & Health, University of New South Wales, Randwick, New South Wales Australia; ^3^ Obstetrics and Gynaecology The Royal Hospital for Women Randwick New South Wales Australia

**Keywords:** birth, breech, education, reality, virtual

## Abstract

**Background:**

Although there is ongoing debate, the current consensus is that vaginal breech birth carries a marginal increase in perinatal morbidity and mortality. Due to these risks there have been decreasing numbers of vaginal breech births and subsequently clinical exposure to hands‐on training has declined. However, to confidently care for women who plan a vaginal breech birth or those presenting in advanced labour with an unexpected breech presentation, education in vaginal breech birth remains necessary.

**Aims:**

This pilot study aimed to assess the ability of a multimodal teaching program consisting of high‐fidelity physical models, educational videos and a 360° virtual reality video to increase the confidence of maternity staff in their theory and management skills regarding vaginal breech birth. A secondary aim was to determine whether the virtual reality video enhanced learning alongside established techniques.

**Materials and Methodology:**

A multimodal teaching program was administered to 20 maternity health staff. They were given a self‐reported pre‐ and post‐intervention scales to assess changes in their confidence. They also provided feedback on the virtual reality video.

**Results and Conclusion:**

The teaching program significantly increased maternity staff's confidence in their knowledge and management skills whilst decreasing their anxiety surrounding vaginal breech birth. However, participants did not perceive the 360^o^ virtual reality being of added value. Further studies should examine whether this program leads to objective change in vaginal breech birth knowledge and management skills and ultimately improved clinical outcomes. Additional studies should explore which types of virtual reality technology benefit breech birth education.

## 1 Introduction

**FIGURE 1:**
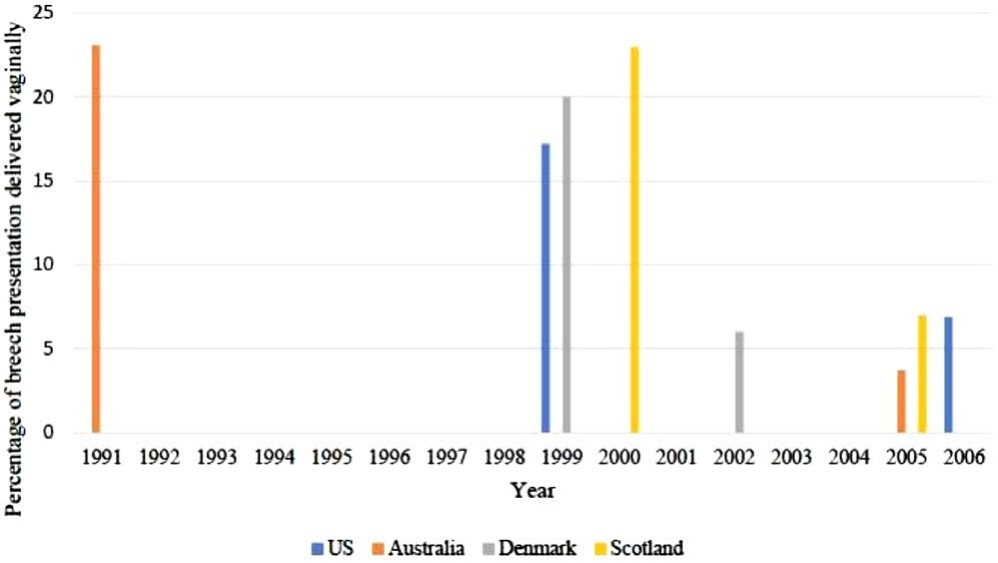
Percentage of breech presentation delivered vaginally by and country. Data adapted from (2, 4–6).

In Australia, approximately 4% of babies are in breech presentation at the time of birth [1]. Breech presentation is where the baby's buttock or feet are facing down the birth canal. The past 20 years have seen decreasing skills and increasing anxiety about vaginal breech birth (VBB) as a result of the Term Breech trial which reported that caesarean section [2] had a lower risk of perinatal mortality, neonatal mortality and neonatal morbidity compared with planned VBB [3]. This has led to the infrequent occurrence of VBB, planned or unplanned. This trend has resulted in fewer opportunities for clinicians to gain experience with VBB, further reducing training opportunities for future clinicians. Figure 1 [2, 4, 5, 6] demonstrates the dramatic reduction in vaginal deliveries in four countries and accounts for the diminished training opportunities in VBB. Education remains necessary to maintain VBB proficiency for the care of women presenting in labour with unexpected breech presentation, and for women who plan a VBB.

An Australian study demonstrated that only 48.1% of trainee obstetricians felt confident with VBB and they did not intend to offer VBB due to potential medicolegal consequences and adverse outcomes further highlighting the fear of litigation [7]. Shoulder dystocia is an example of a previously poorly managed obstetric emergency where the establishment of formal guidelines and use of standardised manoeuvres has led to improved neonatal outcomes [8]. A similar process is needed to assist in VBB management.

Simulation training has been demonstrated in literature to create a safe environment for learning, improving teamwork and patient outcomes and this has also been demonstrated specifically for training of obstetric emergencies [9, 10]. Virtual reality (VR) and augmented reality (AR) could improve the fidelity of current simulation used in VBB training, allowing health professionals to experience it in a format that simulates reality as closely as possible. Two studies, showed students using VR excelled over traditional paper‐based and video learning, demonstrating that VR/AR excels in complex spatial awareness [11, 12]. However, there are also studies that have found no difference between the VR and physical simulation groups in learning intravenous catheter skills and neuroanatomy, indicating that VR technology may not yet be capable of surpassing the benefits of more traditional simulation models [13, 14]. The conflicting evidence on the efficacy of VR/AR in medical education suggests that the type of technology must be tailored to the content being taught.

The current professional consensus is that VBB, even in ideal circumstances, increases perinatal mortality and short‐term neonatal morbidity [15, 16, 17, 18]. Training in VBB remains necessary for the care of women with unexpected breech presentation in labour and for those women who choose to have a planned VBB [19]. Given the rarity of VBB, teaching in this area would further benefit from better access to the skills of a more experienced practitioner along with the use of interactive technology. Hence, using new interactive 360° VR video technology and combining it as an education package with educational videos and high‐fidelity physical models could improve access to and the effectiveness of VBB education, allowing practitioners to feel more prepared to offer women VBB as a service.

## 2 Aim & Hypothesis

The primary hypothesis of this pilot project is that a multimodal teaching program to increase the confidence of maternity staff in their theoretical understanding and management skills concerning VBB is feasible. The secondary hypothesis is that 360° VR video technology will enhance learning alongside more established methods.

Therefore, the aim of this pilot study is to assess the ability of a multimodal teaching program consisting of higher fidelity physical models, educational videos and 360° VR video to increase the confidence of maternity staff in their theoretical understanding and management skills concerning VBB. A secondary aim is to assess the capacity of the 360° VR video in enhancing learning in addition to the above established techniques.

## 3 Materials and Methods

Ethics was submitted to South Eastern Sydney Local Health District HREC (Application ID: 2019/ETH00616) and was deemed a low‐risk study exempt from full ethics approval. Twenty participants were recruited through verbal expressions of interest. Participants had to be an actively practising maternity care employee of the Royal Hospital for Women, consenting, and willing to participate with another colleague. Participants were excluded if they considered themselves highly experienced with VBB.

The 360° VR video was filmed using the ModelMed Sophie and Sophie's mum models simulating the process of normal and complicated VBB. Two 360° video cameras were set up with one filming the viewpoint of the uterus and one filming the birthing process and accoucheur manoeuvres. These videos were then edited so that it could be viewed as a regular 360° video or through a VR headset.

The 20 participants were separated into pairs ideally with medical officers being paired with midwives. Prior to the first phase of the study, participants were asked to complete a Likert‐type confidence scale assessing their current confidence towards their VBB knowledge and management skills.

The first phase of the study involved 90‐min hands‐on teaching sessions by an experienced obstetrician with pairs of participants using the basic baby and pelvis models, the ModelMed Sophie and Sophie's Mum models, and educational breech videos. The second phase of the study involved 30‐min review sessions with pairs of participants using a basic baby and pelvis models, the Laerdal Prompt Flex baby and ModelMed Sophie's Mum models. Participants were also shown a 360° VR video of a normal VBB using a VR headset and provided feedback via a semi‐structured interview. The third phase involved participants being assessed using the global assessment scale simulated emergency VBB scenario. Each item on the assessment was marked out of 5 alongside a global score out of 5. Following this test, the participants were asked to fill in the confidence scale again. The clinical scenarios taught and assessed are attached in Appendix S1.

## 4 Data Analysis

Quantitative analysis was done using IBM SPSS Statistics 25 and qualitative analysis was completed manually. A Wilcoxon Rank‐Sign test was conducted comparing each item of the pre‐intervention confidence scale with the post‐intervention confidence scale. This was reported for the occupations combined due to the occupation group sizes being too small to interpret significance. A Mann–Whitney *U* test was conducted to compare the pre‐ and post‐intervention confidence scale item scores by occupation. A *p* value < 0.05 for all above tests was considered significant.

A summary of the interview responses to the VR video was also provided.

## 5 Results

The 20 participants recruited included five residents, three registrars, eleven midwives and one final year midwifery student. All participants completed Phase 1, 19 participants completed Phase 2, and 12 participants completed Phase 3. These twelve included two registrars, three residents and seven midwives. The loss to follow‐up was due to scheduling conflicts. Only 10 participants watched and provided feedback on the 360° VR video because of technical issues with the video. Due to the limited sample size and the pilot nature of this study, these results are purely exploratory and proof of concept for a larger study.

There was a statistically significant improvement in all confidence scale items relating to the maternity health staff's confidence in their knowledge about the processes and management for both normal and abnormal VBB following the teaching package (Table 1). There was a statistically significant decrease in participants' anxiety towards encountering an unexpected VBB. One participant had a decrease in their confidence towards managing delays with the baby's head.

**TABLE 1:** Wilcoxon Rank‐Sign test comparing post‐intervention confidence scale scores to pre‐intervention confidence scale scores.

Item	Pre‐intervention median (IQR)	Post‐intervention median (IQR)	Significance (two‐tailed *p* value^a^)	Effect size
1. Knowledge of normal breech birth	4 (3, 4.75)	6 (5, 6)	0.002	0.58
2. Knowledge of possible breech complications	4 (3, 5)	5 (5, 6)	0.002	0.58
3. Facilitating a normal breech birth	2 (2, 4)	5.5 (5, 6)	0.001	0.60
4. Identify position of arms when baby not progressing	2 (1, 3)	5 (4, 5.75)	0.001	0.60
5. Ability to manoeuvre baby to release arms to deliver baby	2 (2, 3)	5 (4, 5.75)	0.001	0.60
6a. Anxiety with spontaneous undiagnosed breech born to umbilicus not progressing	6 (5, 6)	4 (4, 5)	0.002	0.59
6b. Ability to identify cause of delay in above situation	4 (2.25, 4)	5 (4.25, 5.75)	0.004	0.55
6c. Ability to manage above situation	2 (1, 3)	4 (4, 5)	0.001	0.61
7. Recognise delay in birth of head	3.5 (2, 4)	5 (5, 6)	0.001	0.60
8. Ability to manage delay in birth of head	2 (1, 3)	5 (4, 5)	0.013	0.50
9. Attending breech birth without supervision	1 (1, 2)	4 (3.25, 5)	0.002	0.58
10. Knowledge of maternal positioning in breech birth	4 (3.25, 4.75)	5 (5, 6)	0.002	0.59

^a^ *p* value < 0.05 considered significant.

Midwives had statistically significantly higher confidence in how maternal positioning facilitates VBB compared with doctors prior to the teaching package (*p* = 0.02). This difference between doctors and midwives was no longer present following the teaching package (*p* = 0.39) with an increase in doctors' confidence in this area. There was no statistical significance between doctors and midwives for all other pre‐intervention confidence scale measures (Table 2). Midwives had statistically significantly higher confidence in their knowledge about the processes in a normal VBB compared with doctors following the teaching package (*p* = 0.01). There was no statistical significance between doctors and midwives for all other post‐intervention confidence scale measures (Table 3).

**TABLE 2:** Mann–Whitney U test comparing pre‐intervention confidence scale scores of midwives and doctors.

Item	Midwives			Doctors			Test statistics	
	*n*	Mean rank	Sum of ranks	*n*	Mean rank	Sum of ranks	Mann–Whitney *U*	Exact sig. (2‐tailed *p* value^a^)
1. Knowledge of normal breech birth	12	9.75	117.00	8	11.63	93.00	39.00	0.51
2. Knowledge of possible breech complications	12	10.29	123.50	8	10.81	86.50	45.50	0.89
3. Facilitating a normal breech birth	12	10.00	120.00	8	11.25	90.00	42.00	0.66
4. Identify position of arms when baby not progressing	12	9.83	118.00	8	11.50	92.00	40.00	0.57
5. Ability to manoeuvre baby to release arms to deliver baby	12	10.17	122.00	8	11.00	88.00	44.00	0.77
6a. Anxiety with spontaneous undiagnosed breech born to umbilicus not progressing	12	10.50	126.00	8	10.50	84.00	48.00	1.00
6b. Ability to identify cause of delay in above situation	12	10.75	129.00	8	10.13	81.00	45.00	0.86
6c. Ability to manage above situation	12	11.00	132.00	8	9.75	78.00	42.00	0.65
7. Recognise delay in birth of head	12	11.83	142.00	8	8.50	68.00	32.00	0.23
8. Ability to manage delay in birth of head	12	9.71	116.50	8	11.69	93.50	38.50	0.46
9. Attending breech birth without supervision	12	11.75	141.00	8	8.63	69.00	33.00	0.23
10. Knowledge of maternal positioning in breech birth	12	12.63	151.50	8	7.31	58.50	22.50	0.02

^a^ *p* value < 0.05 considered significant.

**TABLE 3:** Mann–Whitney *U* test comparing post‐intervention confidence scale scores of midwives and doctors.

Item	Midwives			Doctors			Test statistics	
	*n*	Mean rank	Sum of ranks	*n*	Mean rank	Sum of ranks	Mann–Whitney *U*	Exact sig. (2‐tailed *p* value^a^)
1. Knowledge of normal breech birth	7	8.50	59.50	5	3.70	18.50	3.50	0.01
2. Knowledge of possible breech complications	7	6.57	46.00	5	6.40	32.00	17.00	1.00
3. Facilitating a normal breech birth	7	7.93	55.50	5	4.50	22.50	7.50	0.09
4. Identify position of arms when baby not progressing	7	6.21	43.40	5	6.90	34.50	15.50	0.89
5. Ability to manoeuvre baby to release arms to deliver baby	7	6.57	46.00	5	6.40	32.00	17.00	1.00
6a. Anxiety with spontaneous undiagnosed breech born to umbilicus not progressing	7	4.79	33.50	5	8.90	44.50	5.50	0.06
6b. Ability to identify cause of delay in above situation	7	7.14	50.00	5	5.60	28.00	13.00	0.70
6c. Ability to manage above situation	7	6.43	45.00	5	6.60	33.00	17.00	1.00
7. Recognise delay in birth of head	7	7.43	52.00	5	5.20	26.00	11.00	0.29
8. Ability to manage delay in birth of head	7	7.43	52.00	5	5.20	26.00	11.00	0.41
9. Attending breech birth without supervision	7	7.43	52.00	5	5.20	26.00	11.00	0.37
10. Knowledge of maternal positioning in breech birth	7	7.36	51.50	5	5.30	26.50	11.50	0.39

^a^ *p* value < 0.05 considered significant.

The time taken between administering Phases 1 and 2 ranged from 7 to 63 days with a mean number of 32.71 ± 16.73 days. The time taken between administering Phases 2 and 3 ranged from 1 to 26 days with a mean number of 13.83 ± 9.31 days.

Ten participants provided feedback on the 360° VR video. The comments around the use of VR focused on three main aspects: the role of VR in concentration and capturing attention, the importance of the role of VR in accessible teaching and the use of 360° VR video as a teaching tool.

There was unanimous agreement that the VR technology was novel and immersive however some found the novelty was distracting whilst others found the immersive nature enhanced their concentration as it blocked other distractions.

Most participants agreed that the VR video was a good theoretical knowledge resource for people who had no access to in‐person teaching, however, all agreed that hands‐on experience with a model was necessary, with one response stating that the VR video was ‘futile’ without the accompaniment of hands‐on teaching. The VR aspect also made the video less accessible, and some found the VR technology made them nauseous and that this could be a limiting factor.

All the participants found the VR video to be a valuable reinforcement tool, however they felt the VR component did not significantly enhance their experience. A few participants noted that the VR component would've been more beneficial if it was interactive.

## 6 Discussion

Evaluation of the teaching package demonstrated improvements in the overall confidence of all participants who completed the third phase of the study. It improved their confidence in their theoretical understanding and management skills of VBB and reduced their anxiety towards VBB. This significant increase, despite the limited sample size, could be attributed to the lack of VBB teaching and hence any formal program provides a significant improvement. One participant had a decrease in their confidence towards managing delays in the birth of the head, following the teaching package possibly due to a realisation that these delays were more complex than previously thought.

Similar to another study that found breech management training would be beneficial to facilitating VBB [20], the positive feedback and results about the teaching package suggest that implementing a proper VBB training program similar to that done with shoulder dystocia [8], could facilitate more VBB's by reducing fear and anxiety through increasing the skill of all clinicians. This supports the results of a 2020 retrospective cohort study that found simulation training improved the use of appropriate manoeuvres in VBB [21].

Despite the variation in the timeframes between the phases of the study, all participants appeared to have a significant improvement in their confidence towards their knowledge and management skills about VBB. This study only assesses learner's reaction (Kirkpatrick Level 1) to their experience of the program. A larger study will need to formally assess changes in knowledge and skill (Kirkpatrick Level 2), a change in clinician's behaviour in real life settings (Kirkpatrick Level 3) and ultimately an improvement in perinatal morbidity and mortality (Kirkpatrick Level 4) [10].

Midwives recorded significantly higher confidence in their knowledge about normal VBB following the teaching package, when compared with doctors. This could be because the doctors in this study were less experienced whereas the midwives had been working for more years.

Unexpectedly, participants did not perceive the 360° VR video adding value to their learning experience. Participants unanimously agreed that there would have been no difference if it was a regular video. The inconsistency of VR's effectiveness in medical education is reflected by conflicting evidence reported in current literature; this study further supports the idea that the specific VR technology must be matched to the content being taught. The 360° VR video was from two static points‐of‐view, however both these visual perspectives were distant and obscured which likely decreased the efficacy. Participants also felt that interactive VR technology would have been more effective. To develop a VR model for the teaching of practical skill, there not only needs to be a clear view of the processes but also the ability to manipulate the environment.

The small sample size and pilot nature of the study allowed individualised interaction with participants which helped researchers understand how to develop a more effective teaching program. A single experienced obstetrician delivered all the teaching materials which provided more consistency. The other strength of the study was the use of higher fidelity models.

A clear limitation is both the small sample size as well as the location from which the sample was drawn. This however is fitting with the pilot nature of this study and proves the feasibility of such a program. Working at a hospital that conducts VBB's also likely affects the receptiveness of the participants towards VBB which may have modified the effectiveness of the teaching program and ideally, future studies would draw from varying levels and types of hospitals as well as from differing locations ranging from developing to developed countries.

There was a large amount of variability in the timing between the phases of the study within the participants. Although this did not seem to affect the self‐assessed confidence, it could have had an impact on the true effectiveness of the teaching program since the timing between acquisition of skill, revision and testing affects retention of information [9].

VR models can be used effectively in medical education and especially when the real scenario is dangerous, rare or both. This study revealed it did not effectively utilise VR technology for the content that was being taught although there is a great deal of potential in this field. There are drawbacks with VR/AR due to nausea [22] which was also noted in our study.

A multimodal teaching program to increase the maternity staff's confidence in their theoretical understanding and management skills concerning VBB is feasible however the 360^o^ VR video technology used in this study did not enhance learning in addition to previously established methods. This pilot study is proof of concept that such a teaching program is effective. Further studies should examine whether the teaching program objectively increases VBB knowledge and management skill, and whether the program leads to true behavioural changes and improved clinical outcomes. Further studies should also explore what forms of VR/AR technology would benefit VBB education.

## Conflicts of Interest

The authors declare no conflicts of interest.

## Supporting information


Data S1.
