# Supplementary material for: The Use of 360° VR Video, Educational Videos, and High‐Fidelity Physical Models in Teaching Breech Birth ‐ A Pilot Feasibility Study
Source: Aust N Z J Obstet Gynaecol. 2025 May 7;65(6):831–7. doi: 10.1111/ajo.70040 (PMC12794779; doi:10.1111/ajo.70040)
Supplement: Supplementary file 1 — Data S1. [file AJO-65-831-s001.docx]

## Appendix 1: Clinical scenarios included in teaching program


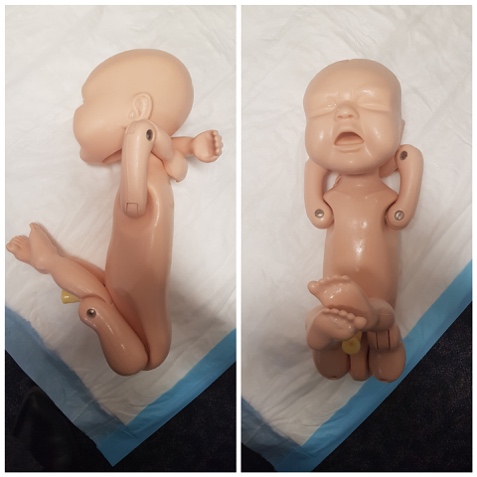

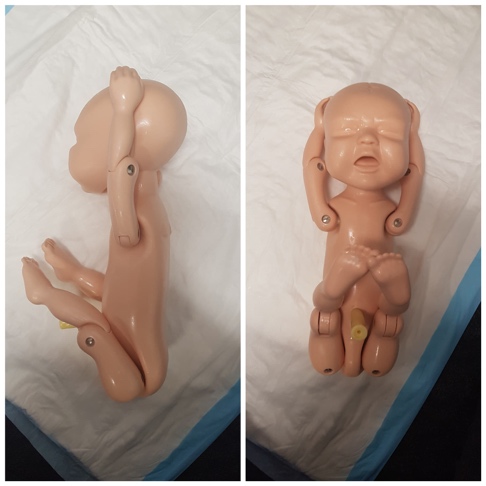

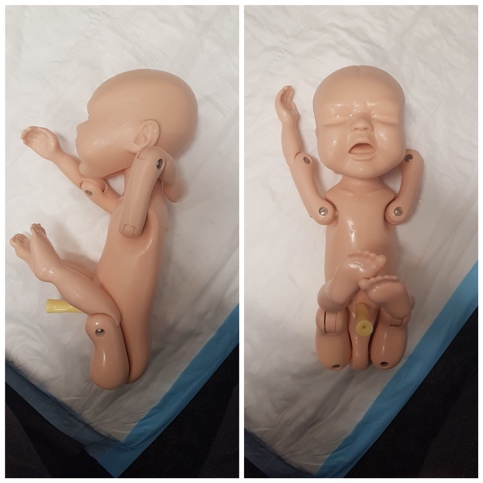


Figure 4: Arms 3 (bilateral extended arms)

Figure 5: Arms 4 (unilateral nuchal arm)

Figure 6: Arms 5 (bilateral nuchal arms)


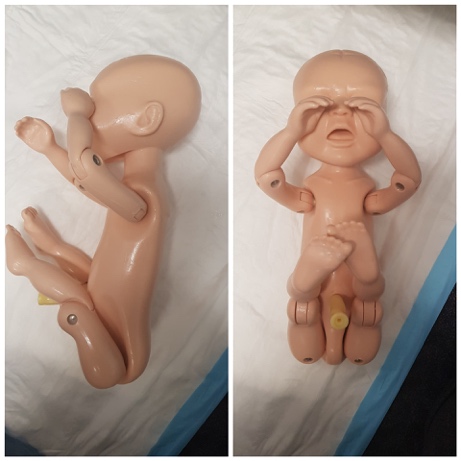

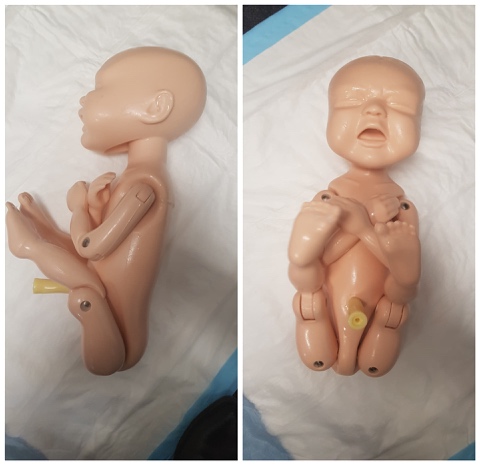

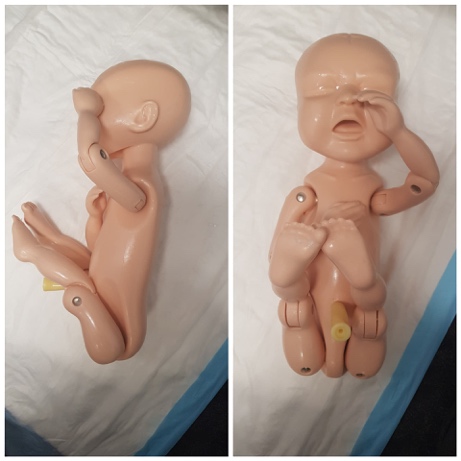


Figure 1: Baby's position in normal frank breech birth

Figure 2: Arms 1 (unilateral hand next to face)

Figure 3: Arms 2 (bilateral hand next to face)
